# Open-Source Telemedicine Platform for Wireless Medical Video Communication

**DOI:** 10.1155/2013/457491

**Published:** 2013-03-13

**Authors:** A. Panayides, I. Eleftheriou, M. Pantziaris

**Affiliations:** ^1^Department of Computer Science, University of Cyprus, 1678 Nicosia, Cyprus; ^2^Department of Electrical and Electronic Engineering, Imperial College, London SW7 2AZ, UK; ^3^The Cyprus Institute of Neurology and Genetics, 1683 Nicosia, Cyprus

## Abstract

An m-health system for real-time wireless communication of medical video based on open-source software is presented. The objective is to deliver a low-cost telemedicine platform which will allow for reliable remote diagnosis m-health applications such as emergency incidents, mass population screening, and medical education purposes. The performance of the proposed system is demonstrated using five atherosclerotic plaque ultrasound videos. The videos are encoded at the clinically acquired resolution, in addition to lower, QCIF, and CIF resolutions, at different bitrates, and four different encoding structures. Commercially available wireless local area network (WLAN) and 3.5G high-speed packet access (HSPA) wireless channels are used to validate the developed platform. Objective video quality assessment is based on PSNR ratings, following calibration using the variable frame delay (VFD) algorithm that removes temporal mismatch between original and received videos. Clinical evaluation is based on atherosclerotic plaque ultrasound video assessment protocol. Experimental results show that adequate diagnostic quality wireless medical video communications are realized using the designed telemedicine platform. HSPA cellular networks provide for ultrasound video transmission at the acquired resolution, while VFD algorithm utilization bridges objective and subjective ratings.

## 1. Introduction

Driven by technological advances, especially in the last decade, mobile-health (m-health) systems and services have refined access to specialized healthcare delivery [1–5]. Advances in wireless and sensor networks, mobile and cloud computing, compression technologies, mobile devices and nanotechnologies, and associated standards and algorithms for efficient communication, interoperability, and ease of integration have fostered the evolution of such systems and services. Toward this end, social media colossal acceptance linked with an overwhelming number of smartphone medical-oriented applications is expected to bring further growth, initiating a decisive subject involvement. While economic benefit is still debatable based on current deployment [6], it is indisputable that widespread adoption in daily clinical practice will provide significant financial savings [7].

**Figure 1:**
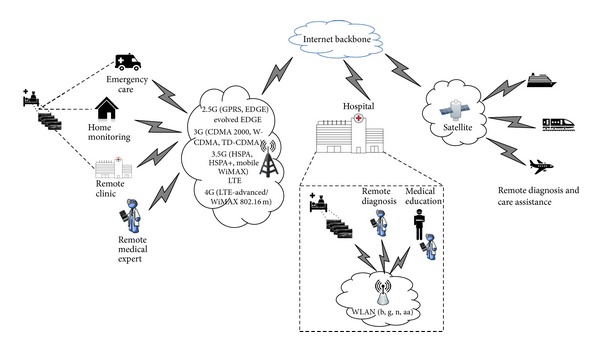
Typical scenarios for m-health medical video communication systems. Medical video is wirelessly transmitted based on the best available wireless network from the patient's side to the medical expert's end for remote diagnosis.

Medical video communication systems aim to meet the demand for emergency telematics, within ambulance care, remote diagnosis and care for frail elderly people and people with mobility problems, mass population screening, especially in developing countries and in disaster incidents and battlefields, and for medical education purposes and second opinion provision (see Figure 1) [8]. Medical video communication m-health systems have been primarily based on impressive data rates increase and extended coverage of wireless infrastructure, linked with new compression technologies and high-efficiency low-complexity codec equipment [9]. Moreover, active involvement of medical experts in the design allowed the development of clinically resilient telemedicine frameworks, increasing system's robustness and objective of providing adequate quality video diagnostics [10].

The most prevailing research trends in the design of m-health telemedicine systems are summarized next: (a) medical video *modality-aware* (m-aware) systems, where individual properties of different video modalities guide the encoding, transmission, and evaluation process (e.g., diagnostically relevant encoding paired with unequal error protection of regions of diagnostic interest) [11–15], (b) multilayer and cross-layer optimization systems, which minimize a cost function constructed of different layer parameters such as packet loss rate (PLR), end-to-end-delay and delay jitter, frame objective ratings (e.g., peak signal-to-noise-ration (PSNR), structure similarity (SSIM) index), video resolution, frame rate, and so forth, for optimum performance [16–19], and finally (c) studies which focus on clinical quality assessment protocols and recommendations [10–12, 20].

In this study, the aim is to develop a low-cost telemedicine platform for the wireless communication of adequate diagnostic quality video using open-source technologies. The aim is to depict that open-source software tools can deliver the acceptable performance required for demanding medical video communications, using commercially available infrastructure. In fact, we aim to bridge the gap between theory and practice, by providing a proof-of-concept study which can accelerate the wider deployment of m-health systems in daily clinical practice. Despite the widespread belief by the research community that such systems and services can provide significant time advantages that can prove vital for the patients' health, the adoption in daily practice is rather limited. This is partly due to the relatively limited studies depicting real-life implementation of medical video streaming systems, attributed to the absence of wireless channels that could support video communications at the in-hospital video resolution. The latter was documented for earlier studies in the literature, where limited upload data rates of 3G channels bounded the successfully communicated medical video to quarter video graphics array (QVGA-320 × 240) resolution [21–24].

Here, we employ top performing open-source software, investigate medical video communications at scalable resolutions, for the most common encoding structures found in the literature today, and evaluate their clinical performance for an overwhelming number of cases. We examine two widely available wireless channels, namely, wireless local area network (WLAN) as the benchmark case and 3.5G high-speed packet access (HSPA) as the default utilization scenario. The objective is to demonstrate that high-resolution medical video communication is possible, approaching the clinical standards of in-hospital examination.

To accomplish this task, we summarize the primary objectives of this paper in three different areas.

- Open-source platform for wireless medical video communication: The primary focus of this paper is the development of an open-source platform that will provide for adequate diagnostic quality medical video communications. Such a system will benefit from low-cost development and ease of deployment especially for telemedicine services in developing countries. Moreover, it will serve as a research tool that will accelerate research in m-health video communication systems and facilitate medical education purposes.
- Coding efficiency: we examine different video resolution transmission for the most widely used coding structures today, employing the most efficient H.264/AVC encoder, namely, x264. The use of higher video resolution such as 560 × 416 resolution suggests that medical video communication at the in-hospital-acquired resolution is possible using open-source technologies.
- Video communications over HSPA networks: we investigate currently available 3.5G mobile cellular networks performance in Cyprus using Quality of Service (QoS) metrics (such as packet loss rate, delay, and delay jitter). The utilization of variable frame delay (VFD) algorithm as a calibration step before computing PSNR aims to bridge the gap between objective and subjective ratings, by removing temporal mismatch between transmitted and received videos.

The rest of the paper is organized as follows: Section 2 provides an overview of the developed open-source platform and outlines individual components characteristics that relate to video transmission.  Section 3 describes the undertaken methodology, while Section 4 provides the experimental evaluation. Finally, Section 5 gives some concluding remarks.

## 2. Open-Source Platform for Wireless Telemedicine

Open-source software is quickly becoming the designated tool for development and evaluation in research and academia, as well as pilots, for potentially commercial applications. This is mainly attributed to the lower costs involved in developing and maintaining open-source applications, source-code availability which enhances reusability and enables customization, and an ever-increasing community which supports and expands available features. Here, the choice of open-source tools aims to develop, evaluate, and disseminate a low-cost telemedicine system, easily deployable, which will provide reliable communication of real-time medical video for remote diagnosis purposes. Such a system can serve as a research tool for the design and development of new m-health systems and services, be used as a medical education tool, and can serve as a telemedicine platform, especially in developing countries. 

**Figure 2:**
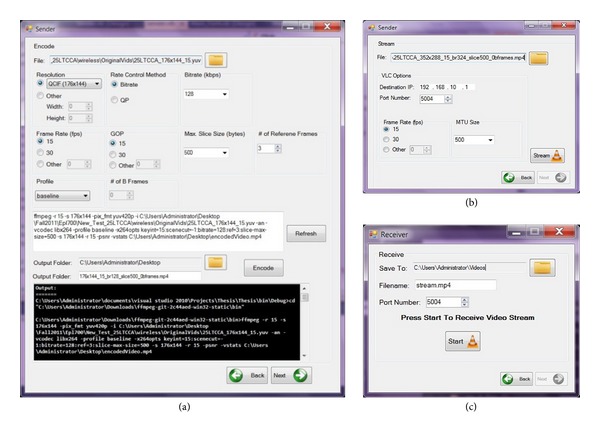
Screenshots depicting the open-source-based medical video communication platform. (a) Encoding interface to the x264 codec. Additional parameters can be inserted via the command line text box situated in the middle of the interface, (b) streaming interface at the sender's side using the VLC media player, and (c) receiver's interface at the receiver's side.

The proposed system's interface is demonstrated in Figure 2. It is worth noting here that the particular interface is the developer's interface. For integration into standard clinical practice, a simpler interface suitable for clinicians will be available. Next, we provide a step-by-step analysis of the system's utilization and the associated open-source components. Following video acquisition, the video is encoded using the FFmpeg software [25] and more specifically the x264 [26] libraries, which implement the H.264/AVC standard. It is worth noting that x264 has been ranked as the most efficient codec in comparative evaluations of widely available H.264/AVC codecs [27]. As illustrated in Figure 2(a), the main encoding features can be selected using the encoding menu, while additional advanced parameters may be inserted using the command line interface. Using VLC player, of the VideoLan project [28], the resulting real-time transport protocol (RTP) packets are streamed over the underlying wireless network to the medical expert's remote end. This procedure is performed using the sender's video communication interface appearing in Figure 2(b). The user is asked to define the receiver's IP address and communication port, in addition to the transmitting frame rate and packet size (if different from the encoding parameters). Simultaneously, Wireshark network protocol analyzer [29] is triggered at the receiver's side for packet monitoring and QoS measurements. The receiver also uses VLC to render the transmitted video, which is decoded using FFmpeg. At the receiver's side, only the incoming port needs to be defined (see Figure 2(c)). For video quality assessment purposes, the received video is stored at the remote end. The latter enables full reference (FR) objective video quality assessment (VQA) algorithms such as PSNR to validate the capacity of the proposed system to accommodate adequate diagnostic quality medical video communications. 

## 3. Variable Frame Delay

The variable frame delay (VFD) [30] algorithm has been recently introduced by the National Telecommunication and Information Association (NTIA). VFD aims to alleviate the temporal mismatch between transmitted and received video frames, likely to be introduced by video pauses during video transmission. Such pauses can occur due to varying network state, resulting from signal attenuation, mobility, handover, and so forth. To compensate for these changes, adaptive streaming algorithms may employ temporal downsampling of the streaming content, which is a common practice in scalable video streaming and cross-layer design systems, or even choose to drop frames. Moreover, frame freezing (displaying the previous frame in the absence of the current frame) is a widespread error concealment method found in many codecs. As a result, full reference VQA metrics often fail to deliver ratings that do correlate with perceptual quality. VFD, acting as a calibration step before FR quality assessment, removes temporal mismatch and allows objective FR algorithms to provide reliable quality measurements.

Variable frame delay algorithm computes the mean square error (MSE) between the normalized processed (received) frame and a predefined window of normalized original frames, for a given region of interest. In this manner, when it comes to computing the objective VQA metric, the algorithm uses for comparison the original frame that minimizes the MSE with the relevant processed frame.

Provided that all computations are performed using only the luminance information (or the *Y* channel), video sequences for the following formulas will be denoted as *Y*(*i*, *j*, *t*), where *t* = 0, 1, 2, …, *N* − 1, *N* is the total number of frames parting the video, and *i* and *j* correspond to the image's row and column, respectively. A subscript *p* denotes a processed video (i.e., transmitted video), while a subscript *o* stands for the original, uncompressed video sequence. The procedure for normalizing processed video frames to have zero mean and unit variance appears in (1):

(1)   Yp(i,j,tp)=(Yp(i,j,tp)−mp)σp,(i,j)∈SROI,  tp=0,1,2,…,N−1.

SROI stands for spatial region of interest. This is a user-defined parameter which aims to restrict frame alignment computations within the most significant video region as well as eliminate boundary pixel values. Similarly, the original video is normalized using

(2) Yo(i,j,to)=(Yo(i,j,to)−mo)σo, (i,j)∈SROI,to=firstalign+firstalign+1, firstalign+2,…,firstalign+N−1.

Compared to the processed video sequence, the video time durations used for mean and standard deviation computation for the original video sequence use the term first_align_, which corresponds to the best match guess between processed and original frames. Next, having normalized both sequences, the MSE between processed and original videos for a (user) predefined window of frames is estimated using (3). This window is denoted as temporal uncertainty (*t*_uncert_). When not all frames are available (e.g., beginning or end of each sequence), only the available frames are used for computing the MSE:

(3) MSE(to,tp)=meanover  i,j{[Yo(i,j,to)−Yp(i,j,tp)]2},tp=0,1,2,…,N−1,to=firstalign−tuncert+tp,…,firstalign, +tp,…,firstalign+tuncert+tp.

To reduce alignment errors the algorithm imposes an additional causality constraint, based on heuristic methods. However, the techniques used during these steps of the VFD algorithm are outside the scope of this paper. A detailed analysis of the algorithm's components and implementation in MATLAB appears in [30]. VFD algorithm can be used both as a calibration step before employing a FR VQA algorithm and for estimating the impact of temporal mismatch caused during real-time video streaming. 

## 4. Methodology

### 4.1. Encoding Setup

Five atherosclerotic plaque ultrasound videos, with spatial video resolution of 560 × 416 acquired at 40 frames per second (fps), compose the dataset used during the experimental evaluation. The investigated encoding setup includes scalable video resolutions that reflect the most common resolutions used in atherosclerotic plaque ultrasound video transmission, different bitrates according to the wireless channel's capacity, and different encoding schemes and profiles for improved efficiency.  Table 1 summarizes the encoding parameters that were used in this study.

For the lower QCIF resolution (176 × 144), the videos were encoded at the following bitrates: (a) 128 kbps, (b) 256 kbps, and (c) 324 kbps. These bitrates are well within the typical upload data rates of 3.5G high-speed downlink packet access (HSDPA, Rel. 5 [31]) wireless networks. For the CIF resolution (352 × 288), in addition to the aforementioned bitrates, ultrasound videos were also encoded at 512 kbps, to ensure adequate diagnostic quality. Finally, videos at the acquired, 560 × 416 resolution were encoded at bitrates of 512 kbps and 768 kbps. High-speed uplink packet access (HSUPA, Rel. 6 [32]) mobile cellular networks can accommodate upload transmission rates of the latter two resolutions.

To investigate the most efficient encoding structure, we examined the most common encoding formats used in real-time video transmission found in the literature today. More specifically, we employed IPPPP, IPBBP, IPBBBBP, and IPBBBBBBBBP schemes. The main H.264/AVC profile was used for all schemes besides the IPPPP case, which was encoded using the baseline profile.

The values of the remaining encoding parameters were selected using coarse-to-fine parameter optimization for the parameters appearing in the literature. The maximum slice size is set to 500 bytes, as this value was found to minimize latency and provided for better objective ratings compared to higher values. The packet size was selected accordingly, so that when a packet is lost or corrupted, the error would be limited within slice boundaries. GOP size and intra-update interval is set to fifteen frames. In this manner and in conjunction with a frame rate of 15 fps, error propagation extends to at most one second. This is very important for medical video communications, as it maximizes the probability of error-free cardiac cycles, hence clinical quality.

### 4.2. Investigated Scenarios

#### 4.2.1. Wireless LAN Medical Video Communication

The system's performance is demonstrated based on two typical wireless transmission scenarios. The first scenario examines medical video communications within a wireless local area network (WLAN). This scenario provides for medical video transmission within the hospital facilities. Anticipated integration of such systems in daily clinical practice is expected to reduce in-hospital delays, potentially unsafe for the patient's health movements for routine exams, as well as medical expert's visits to outpatient clinics. Moreover, it can be used for educational purposes in university hospitals. This can be considered as the benchmark scenario.

#### 4.2.2. 3.5G HSPA Medical Video Communication

The second scenario investigates wireless communications based on commercially available high speed packet access (HSPA) network in Cyprus. Despite a theoretical upload speed of 5.8 Mbps advertised by the provider, the typical measured upload speed is around 1 Mbps, with an average delay of 135 ms. The sender is connected to the 3.5G network, while the receiver is connected to a WiFi local network. Similarly to the first scenario, the measurements took place in different time periods for a realistic approximation of actual conditions. This scenario simulates medical video transmission in emergency situations to the hospital and/or from remote areas and generally areas where internet access is only available via a cellular network. Likewise, it can be used for mass population screening purposes and most importantly as a gateway of access to specialized care in developing countries with limited resources.

### 4.3. Video Quality Assessment

To validate the performance of the proposed system, objective and subjective VQA was employed, while quality of service (QoS) measurements assessed network's stability. Objective evaluation was based on the widely used PSNR algorithm. However, as documented in [33], PSNR often fails to correlate with subjective ratings. For that reason, and as documented in Section 3, VFD algorithm was applied as a calibration step before PSNR computations. For the computation of the QoS metrics, like the packet loss rates (PLR) and the end-to-end delay, Wireshark network protocol analyzer was employed.

Subjective (clinical) ratings were based on the clinically established protocol described in [9]. Using a rating scale of 1 to 5, a rating of 5 signified a diagnostically lossless video, while a rating of 4 an acceptable loss of minor details. At the lowest end, a rating of 1 suggested that the transmitted video was of no clinical interest. The rating scale appears in Table 2. The medical expert was asked to provide individual ratings for (a) *plaque presence*, (b) *degree of stenosis*, and (c) *plaque type and morphology*. As documented in [9–11, 20], encoding parameters like video resolution, frame rate, and compression ratio greatly impact the clinical capacity of the transmitted video (see Table 3). More specifically, CIF resolution allows sufficient clinical information for categorizing plaque type, as opposed to QCIF resolution. Similarly, higher 4CIF resolution enables the medical expert to assess plaque morphology, which is not always feasible with lower CIF resolution. 

## 5. Results and Discussion

In this section we discuss the experimental evaluation of the proposed medical ultrasound video transmission platform. We present results in terms of investigated coding structures efficiency, performance of real-time medical video transmission over WLAN and HSPA channels, and clinical capacity of the communicated ultrasound videos. Results were obtained using two Lenovo ThinkPad T500 laptops, Intel Core 2 @ 2.53 GHz, 4G RAM, and 32-bit Windows 7 operating system.

### 5.1. Coding Structures Efficacy

**Figure 3:**
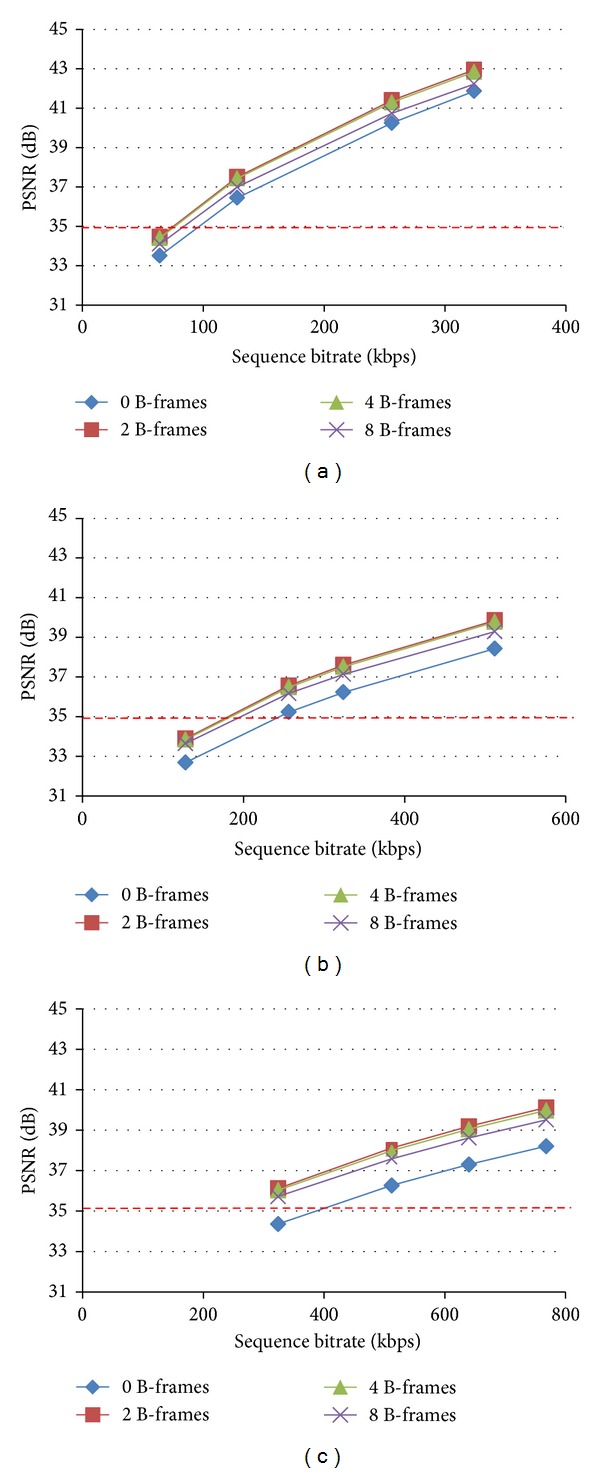
Rate-distortion curves demonstrating coding structure's efficiency at different resolutions and bitrates, for a typical atherosclerotic plaque ultrasound video. (a) QCIF (176 × 144) resolution, (b) CIF (352 × 288) resolution, and (c) 560 × 416 (clinically acquired) resolution.

**Figure 4:**
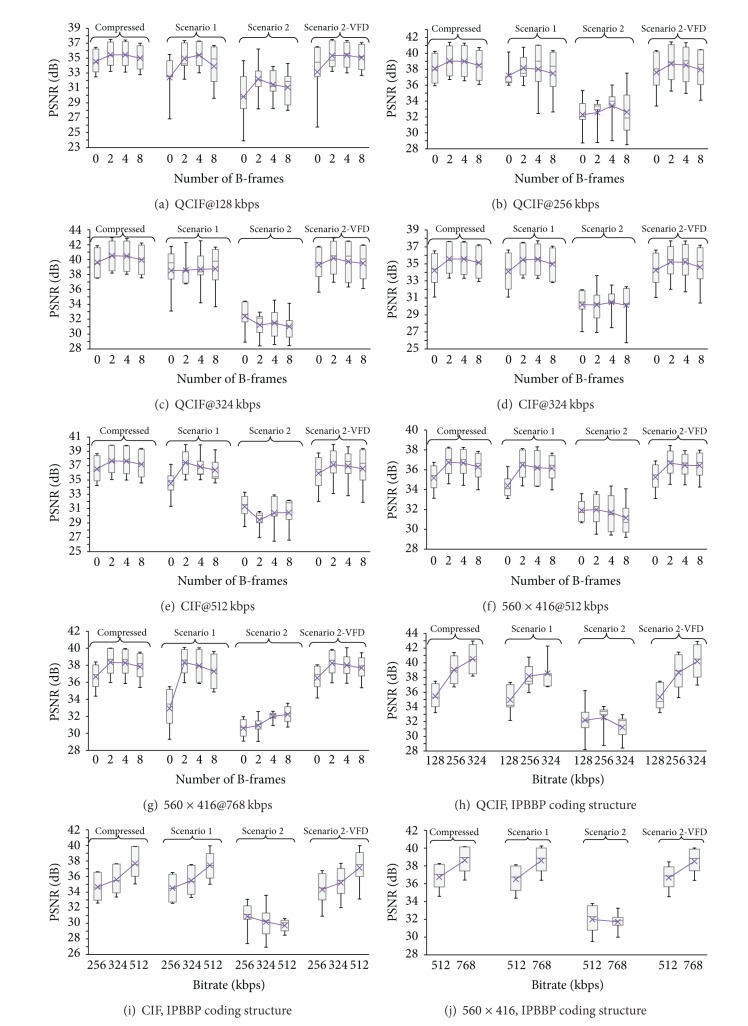
Box plots depicting the PSNR ratings for the five videos parting the examined dataset and the two investigated scenarios. Figures 4(a)–4(g) illustrate the objective scores for all coding structures, resolutions, and bitrates described in the experimental setup, while Figures 4(h)–4(j) summarize the results for the best performing IPBBP coding structure. Removal of temporal mismatch in the received video in scenario 2, using the VFD algorithm, results in high PSNR ratings, compared to that of scenario 1, and in accordance with the clinical evaluation (see Table 6).

For the purposes of this study, four coding structures widely used in video streaming applications today were investigated.  Figure 3 shows coding structures compression performance at different bitrates and video resolutions, for a typical ultrasound video. The trend is the same for all videos parting the dataset as depicted in the left-most box plots of Figures 4(a)–4(g). Unsurprisingly, B-frames utilization increases coding efficiency. Coding structures employing bidirectional prediction depict comparable performance, with IPBBP and IPBBBBP attaining the best result, and IPBBBBBBBBP following closely. On the other hand, single-directional prediction achieves lower compression ratios. However, IPPP encoding benefits from lower motion estimation times and increased error resilience in noisy environments [9, 34].

Denoted by the red-dashed line, is the clinically acceptable threshold for ultrasound video communication described in [11]. For QCIF resolution, all but the lowest investigated bitrates achieve PSNR ratings higher than the designated threshold. For higher CIF resolution, as depicted in Figure 3(b), IPPP coding structure at 256 kbps is marginally above the desired threshold. Therefore, using a higher bitrate of 324 kbps would be more appropriate when streaming clinically important content. Similarly, for the higher 560 × 416 video resolution, single-directional prediction encoding falls below the designated threshold at 324 kbps, while higher investigated bitrates attain diagnostically acceptable ratings. Here, it is important to note that clinical capacity is directly affected by video dimensions (see Section 4 and Table 3). 

### 5.2. Real-Time Medical Video Communications

#### 5.2.1. Scenario 1: Wireless LAN Medical Video Communication

This is the benchmark scenario. Video packets do not traverse outside the controlled wireless environment, which is not extended beyond the hospital premises. As a result, packet end-to-end delay and packet loss rates are minimal, in the order of 50 ms and ≤1%, respectively, resulting in high diagnostic quality ultrasound video rendering. As evident in the *scenario 1* denoted box plots of Figures 4(a)–4(g), for all videos in the examined dataset, objective ratings following wireless transmission are well above the desired threshold for diagnostically lossless communications, approaching the ratings of the compressed video prior to transmission. The latter is also shown in Table 5, where average PSNR ratings of all investigated cases are documented. It is worth noting here that widespread earlier WLAN IEEE 802.11 standards such as a, b, g, and n have not been designed to facilitate video streaming content. However, due the high data rates and strong signal coverage in the vicinity of a building area, they have accommodated such applications, especially in the absence of severe background traffic (avoiding collision and resources overload), as in the presented scenario. The new generation of WLANs, however, and more specifically the IEEE 802.11aa standard [35] termed “Robust streaming of Audio Video Transport Streams,” is expected to mitigate this phenomenon and provide for demanding video streaming content such as medical video.

#### 5.2.2. Scenario 2: 3.5G HSPA Medical Video Communication

The present scenario resembles a realistic implementation of the developed m-health application. The transmitting end is situated kilometers away from the hospital premises, while varying network conditions affect the quality of the transmitted stream. Figures 4(a)–4(g) depict the box plots of PSNR ratings of the received video (denoted as *scenario 2*).

The objective quality drops abruptly (see also Table 5), failing to match the clinically acceptable threshold. This is partly attributed to the increased delay and packet loses. However, neither the end-to-end delay of 135 ms nor the PLR values between 1-2% are high enough to justify such a dramatic decrease in ultrasound video quality. The latter observation was verified by the clinical evaluation (see below), which provided clinically accepted ratings for the HSPA communicated videos. As the medical expert noted, a temporal freeze in the received video could not compromise the diagnostic capacity of the examined videos, as the clinical information available in prior and subsequent cardiac cycles was sufficient for a confident diagnosis.

Driven by the previously finding and bearing in mind that PSNR often fails to correlate with perceived video quality, led us in considering means of objectively evaluating the instantaneous temporal freeze effect present in the received video. Variable frame delay algorithm was specifically designed to address video pauses during live streaming events, dictated by the high likelihood of such occurrences and the need to evaluate their perceptual impact. As demonstrated in Table 4, low PSNR ratings are attributed to specific video pauses which cause a temporal mismatch between the transmitted and the received video sequences. Table 4 highlights a typical temporal misalignment between the transmitted video (stored at the receiver side) and the original video, used for full reference VQA ratings. Two different sorts of temporal mismatch appear. At frame 17th, there is a one frame skip in the transmitted video, which results either from a lost frame or a delayed frame. In the absence of VFD calibration, FR VQA would propagate this error to subsequent frames as well, resulting in miscomputation of PSNR ratings. The second misalignment appears at the 30th frame. Here, we observe a temporal freeze with a three-frame duration (frames 31 and 32 are the same with frame 30). Again, VFD calibration prevents PSNR measurements to be computed between frames with a temporal mismatch of the three frames. It is worth noting here that temporal mismatch experienced is amplified when using bidirectional prediction, while it is relatively moderate for single-directional prediction (see also Clinical Evaluation section). Applying VFD calibration for removing temporal disparity enables PSRN metric to compare the most relative frames and hence deliver ratings which correspond to the ultrasound video's actual clinical capacity. The right-most box plots of Figures 4(a)–4(g) show that the PSNR ratings following VFD calibration are significantly higher than those prior to VFD calibration and close to those of the transmitted ultrasound video.

Despite the fact that these ratings are in accordance with the clinical evaluation as detailed later, further tuning is required to accommodate a fair objective evaluation. In the presented results, received frames were compared to the most relative transmitted frames indicated by the VFD algorithm, without addressing erroneous and delayed frames causing the temporal mismatch. As a result, the depicted PSNR ratings are based on fewer frames than the total sequence frames resulting in slightly higher PSNR values. This is a matter of ongoing investigation. 

Table 7 demonstrates the results of Kruskal-Wallis (KW) nonparametric analysis of variance (ANOVA) test, which statistically compares the objective results of the investigated transmission scenarios illustrated in Figure 4 and Table 5, for 560 × 416 resolution video. As depicted in Table 7, and in accordance with the aforedescribed scenarios discussion, results obtained using WLAN transmission and HSPA wireless network following VFD calibration are close to the compressed PSNR values prior transmission. As a result, there is no significant difference when statistically comparing these scenarios. On the other hand, HSPA wireless transmission PSNR values prior to VFD calibration are significantly different from the compressed videos PSNR values prior to transmission, as well as the rival wireless transmission schemes, as evident in Table 7. The presented results for the higher 560 × 416 video resolution also hold for the lower QCIF and CIF resolutions.

### 5.3. Clinical Evaluation

 In the context of the clinical evaluation of this work, the physician evaluated a representative sample of the different encoding methods, image analysis, and quality (bitrate). The method used is based on [11] and illustrated in detail in Table 2. The videos were played back on a laptop at their original pixel dimensions. The medical expert was able to access the original video before each evaluation session and provided individual ratings for plaque presence, artery stenosis, and plaque-type and morphology.

As expected, clinical ratings for the first scenario, depicted in Table 6(a), suggest diagnostically lossless communication. More specifically, the clinical information in the transmitted video is equivalent to that of the original video, for detecting atherosclerotic plaque formation on artery walls and estimating the corresponding degree of the artery stenosis. For the most demanding task of plaque type characterization, clinically acceptable ratings (over 4) were assigned in all cases. Naturally, higher quality translates to better ratings. CIF resolution encoded at 512 kbps and 560 × 416 resolution encoded at 768 kbps attained higher scores than CIF resolution encoded at 324 kbps and 560 × 416 resolution encoded at 512 kbps, respectively. Moreover, the best results were obtained for the highest video resolution.

**Figure 5:**
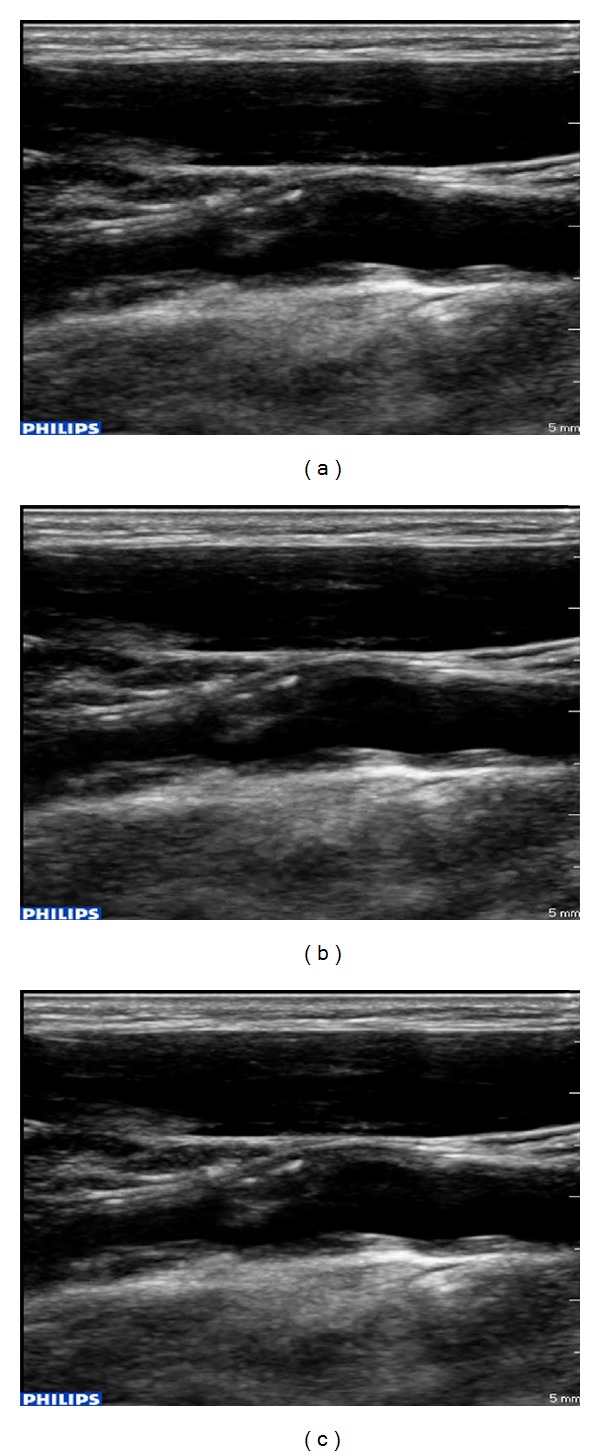
Video image examples of a typical ultrasound video at the acquired, 560 × 416 video resolution. (a) Original ultrasound video, (b) scenario 1 (WLAN): 560 × 416 @ 768 kbps, PSNR: 39.9 dB, and (c) scenario 2 (HSPA): 560 × 416 @ 768 kbps, PSNR-VFD: 39.8 dB.

Clinical evaluation of the HSPA communicated videos appears in Table 6(b). Despite video pauses experienced in the received video, the medical expert is able to provide a confident diagnosis, as evident by the diagnostically acceptable ratings (besides plaque-type assessment for CIF resolution at 324 kbps). Diagnostically lossless cardiac cycles deliver the clinical information needed for proceeding to a diagnosis. Contrary to scenario 1, however, it is not clear that higher resolution and bitrates facilitate higher clinical ratings. While the aforementioned statement is true for CIF resolution, for 560 × 416 resolution the ratings are the same for the investigated bitrates. This is due to the fact that clinical ratings are primarily affected by the frequency of the temporal misalignments. A very important observation commented by the medical expert is that single-directional prediction is more resilient to temporal mismatch, while IPBBBBBBBBP coding structure is more vulnerable to video pauses, among bidirectional prediction investigated schemes. This is attributed to the increased gap between anchor P-frames and the absence of hierarchical coding. The key finding here is that diagnostically acceptable medical video communications at the clinically acquired resolution can be facilitated for the data transfer rates found in HSPA wireless networks (see Figure 5). The latter is not possible for 3G and HSDPA communications, which are restricted by the upload data transfer speeds. CIF resolution medical video communication stands as the boundary scenario for these two wireless networks.

## 6. Concluding Remarks

This paper demonstrates the capacity of the proposed open-source-based telemedicine platform to accommodate reliable wireless communication of medical video. Comprehensive experimentation showed that adequate diagnostic quality ultrasound video at the clinically acquired resolution can be realized using both WLAN and commercially available HSPA wireless networks. The former wireless channel can provide for medical education purposes and remote diagnosis within the hospital premises. The latter can be broadly used for remote access to specialized physicians, in emergency incidents, and for mass population screening. Low-cost implementation and ease of deployment can turn out to be a valuable tool for providing m-health solutions in developing countries, as well as for research purposes. Both objective and subjective video quality assessment methods were employed to validate the clinical capacity of the transmitted video. Bidirectional prediction utilization provided for higher objective ratings than single-directional prediction. Using the VFD algorithm to remove the temporal mismatch present in HSPA communicated video, as a calibration step before FR VQA metrics, enabled computing objective scores that correlate with medical experts ratings. 

Ongoing research includes enhancing the current platform with modality-aware encoding features, investigating mobility aspects in wireless communication for emergency care and extending experiments over long-term evolution (LTE) and LTE-advanced wireless channels. Moreover, mobile devices with limited resources like smart phones and tablets, based on Android and IOS operating systems are currently considered as end-user equipment. Additionally, we also want to investigate how the new high-efficiency video coding (HEVC) standard can lead to more efficient, diagnostically resilient encoding [36]. The proposed framework is currently validated for use in other medical video modalities including trauma, abdominal aortic aneurysm (AAA), and fluoroscopy medical videos.

## Figures and Tables

**Table 1:** Encoding setup.

Parameters	Value	Parameters	Value
Resolution	QCIF (176 × 144), CIF (352 × 288), 560 × 416	Codec	x264
Bitrate (kbps)	128, 256, 324, 512, 768	Profile	Baseline, main
No. of B-frames	0, 2, 4, 6, 8	No. of frames	100
Frame rate, GOP size, intraupdate interval	15	Entropy coding	CABAC
No. of reference frames	3	OutFileMode	RTP
Maximum slice size (bytes)	500	Error concealment	Frame freeze

**Table 2:** Clinical evaluation rating system.

	Plaque detection	Stenosis	Plaque type
5	Plaque(s) presence in transmitted video identifiable as in original	Degree of stenosis in transmitted video determined as in original	Plaque-type classification in transmitted video as in original
4	Plaque(s) presence easily diagnosed	Enough clinical data to determine degree of stenosis	Enough clinical data for plaque-type classification
3	Plaque(s) presence diagnosed, careful attention needed	Clinical data only allow approximation of degree of stenosis	Plaque-type classification is case dependent
2	Plaque(s) presence may be diagnosed after freeze of a clean frame	Very limited ability to estimate degree of stenosis	Not classified
1	Not detectable	Not determinable	Not classified

**Table 3:** Clinical evaluation criteria and associated encoding parameters.

	Clinical significance	Clinical differentiation for	
		Display resolution	Frame rate
Plaque boundary	Diagnose plaque(s) presence and plaque boundary	QCIF (176 × 144), CIF (352 × 288)	≥5 fps
Stenosis	Estimate the degree of stenosis	QCIF (176 × 144), CIF (352 × 288)	≥5 fps Recommended ≥10 fps
Plaque type	Assess plaque morphology and plaque components and determine plaque type	≥CIF (352 × 288)	≥10 fps Recommended 15 fps

**Table 4:** VFD algorithm frame calibration example.

Received video frame no.	*⋯*	15	16	17	18	19	20	21	22	23	*⋯*	30	31	32	33	34	35	36	37	*⋯*
Original video frame no.	*⋯*	15	16	18	19	20	21	22	23	24	*⋯*	31	31	31	32	33	34	35	36	*⋯*

**Table 5:** Video quality assessment measurements (PSNR) for investigated resolutions, coding structures, and bitrates.

No. of B- frames	QCIF					CIF					560 × 416				
	BitRate^a^	Com.^b,c^	WLAN	HSPA	VFD	BitRate	Com.	WLAN	HSPA	VFD	BitRate	Com.	WLAN	HSPA	VFD
0	*128 *	34.5	32.4	29.8	33.1	*256 *	33.6	33.1	30.3	33.2	*512 *	35.2	34.4	31.9	35.0
	*256 *	38.1	37.2	32.3	37.6	*324 *	34.5	34.1	30.2	34.2	*768 *	37	33.6	31.4	36.8
	*324 *	39.6	38.5	32.4	39.3	*512 *	36.5	34.5	30.9	35.9	—	—	—	—	—

2	*128 *	35.4	35.0	32.2	35.0	*256 *	34.7	34.5	30.9	34.3	*512 *	36.7	36.5	32.0	36.5
	*256 *	39	38.1	32.6	38.1	*324 *	35.6	35.5	30.2	35.2	*768 *	38.6	38.6	31.7	38.5
	*324 *	40.5	38.6	31.2	40.1	*512 *	37.7	37.4	29.7	37.1	—	—	—	—	—

4	*128 *	35.4	35.4	31.5	35.4	*256 *	34.6	34.4	31.0	34.3	*512 *	36.7	36.2	31.7	36.0
	*256 *	39	38.1	33.5	38.2	*324 *	35.6	35.5	30.5	35.2	*768 *	38.5	38.2	32.7	38.2
	*324 *	40.5	38.7	31.5	39.2	*512 *	37.6	36.8	30.5	36.9	—	—	—	—	—

8	*128 *	35.0	33.9	31.1	35.0	*256 *	34.3	34.1	30.1	33.9	*512 *	36.3	36.1	31.2	36.0
	*256 *	38.5	37.5	32.6	37.7	*324 *	35.2	35	30.1	34.6	*768 *	38.1	37.6	32.9	37.4
	*324 *	39.9	38.8	31.1	39.2	*512 *	37.2	36.4	30.4	36.5	—	—	—	—	—

^a^Bitrate in kbps, ^b^all PSNR measurements in dB, ^c^compressed video.

**Table:** (a)

No. of B-frames	0	2	4	8
(a) CIF, 324 kbps, scenario 1				

Plaque detection	5	5	5	5
Artery stenosis	5	5	5	5
Plaque-type	4	4	4	4

(b) CIF, 512 kbps, scenario 1				

Plaque detection	5	5	5	5
Artery stenosis	5	5	5	5
Plaque-type	4.2	4.2	4.2	4.2

(c) 560 × 416, 512 kbps, scenario 1				

Plaque detection	5	5	5	5
Artery stenosis	5	5	5	5
Plaque-type	4	4	4	4

(d) 560 × 416, 768 kbps, scenario 1				

Plaque detection	5	5	5	5
Artery stenosis	5	5	5	5
Plaque-type	4.4	4.4	4.4	4.2

**Table:** (b)

No. of B-frames	0	2	4	8
(e) CIF, 324 kbps, scenario 2				

Plaque detection	5	5	5	4
Artery stenosis	5	5	5	5
Plaque-type	4	4	4	3

(f) CIF, 512 kbps, scenario 2				

Plaque detection	5	5	5	5
Artery stenosis	5	5	5	5
Plaque-type	4	4	4	4

(g) 560 × 416, 512 kbps, scenario 2				

Plaque detection	5	5	5	4
Artery stenosis	5	5	5	5
Plaque-type	4	4	4	4

(h) 560 × 416, 768 kbps, scenario 2				

Plaque detection	5	5	5	4
Artery stenosis	5	5	5	5
Plaque-type	4	4	4	4

**Table 7:** Kruskal-Wallis nonparametric analysis of variance test to statistically compare (at *α* ≤ 0.05) the objective results (PSNR) of the investigated wireless transmission scenarios for 560 × 416 resolution videos.

	Com.	WLAN	HSPA	VFD
Com.	—	NS^1^	S^2^	NS
WLAN	NS	—	S	NS
HSPA	S	S	—	S
VFD	NS	NS	S	—

^1^NS designates nonsignificant difference between the compared scenarios.

^2^S designates significant difference between the compared scenarios.
